# MicroRNA-31 mediated inhibition of keratin 6 by PSORI-CM01: a novel approach to psoriasis amelioration

**DOI:** 10.3389/fchem.2025.1636529

**Published:** 2025-07-18

**Authors:** Shilei Wang, Jingkai Wang, Helin Pan, Ruogu Yang, Fanli Zeng, Yongfei Fang, Jinwei Zhang

**Affiliations:** ^1^Chongqing Hospital of Traditional Chinese Medicine, Chongqing University of Traditional Chinese Medicine, Chongqing, China; ^2^Chongqing General Hospital, Chongqing University, Chongqing, China

**Keywords:** psoriasis, miR-31, keratinocyte 6, PSORI-CM01, traditional medicine

## Abstract

**Background:**

Psoriasis vulgaris is a serious noncommunicable disease, with no clear cause or cure. Expression of microRNA-31 (miR-31) is significantly increased in the cutaneous tissue of psoriasis vulgaris patients. Keratin 6 (Krt6) serves as a pivotal biomarker in the diagnostic and therapeutic approaches for psoriasis vulgaris. PSORI-CM01, a traditional Chinese medicine formulation comprising seven medicinal herbs, is employed in China for the therapeutic management of psoriasis vulgaris. However, its anti-psoriatic mechanism warrants further investigations. In this study, the underlying anti-psoriasis mechanism of PSORI-CM01dependent of miR-31 and Krt6 was explored.

**Methods:**

*In vivo*, BALB/c mice were subjected to treatment with imiquimod (IMQ) to establish a psoriasis-like murine model. These psoriasis-like mice were then administered varying concentrations of PSORI-CM01. Following this, evaluations were performed on their Psoriasis Area and Severity Index (PASI) scores, epidermal thickness, and the expression levels of miR-31 and Krt6. HaCaT cells were subjected to treatment with interleukin-6 (IL-6) to create a psoriasis-like cellular model. Following this, the psoriasis-like keratinocytes were administered varying concentrations of PSORI-CM01, and the expression levels of miR-31 were quantified. In addition, these psoriasis-like keratinocytes were transfected with miR-31 mimics and subsequently treated with PSORI-CM01. The expression levels of Krt6 were then quantified and subjected to analysis.

**Results:**

*In vivo*, PSORI-CM01 significantly alleviated the clinical-like manifestations of erythema, scales, and thickening in psoriasis-like mice, and it also reduced the PASI scores; Different concentrations of PSORI-CM01 significantly decreased epidermal thickness and the expression of miR-31 and Krt6 in psoriasis-like mice in a dose-dependent manner. *In vitro*, PSORI-CM01 significantly inhibited the expression of miR-31 and Krt6 in psoriasis-like keratinocytes; However, the decreased Krt6 protein expression was restored by miR-31 mimics.

**Conclusion:**

PSORI-CM01 may improve psoriasis-like lesions by inhibiting expression of Krt6 protein dependent of miR-31.

## 1 Background

Psoriasis vulgaris, with typical clinical characteristics with erythema, scales and thickening of the skin, is a common but complex disease with unknown definitive cause or cure. Psoriasis vulgaris is serious disease with an unpredictable course of symptoms and numerous substantial comorbidities, such as cardiovascular disease, metabolic syndrome, among others. As the 2016 report by the World Health Organization, the global incidence of Psoriasis vulgaris fluctuates between 0.09% and 11.4%, impacting a minimum of 100 million individuals globally (World Health Organization, 2016). This highlights the significance of psoriasis as a major global health concern.

Keratinocytes constitute the principal cellular component of the epidermis, which is the skin’s most superficial layer. Approximately 90% of epidermal skin cells are comprised by keratinocytes in humans. Keratins, as major components of the keratinocytes, is a cytoskeleton protein. The research has shown that elevated expression of keratin 6 is one of the important pathological characteristics of psoriasis vulgaris (Qiao et al., 2022; Yang et al., 2017). MicroRNA (miR) is a non-coding single-stranded RNA molecule that participates in post-transcriptional gene expression regulation. MiR-31 has been observed to exhibit elevated expression in the skin lesions of patients with Psoriasis vulgaris, suggesting its potential role in the pathogenesis and progression of the condition (Yan et al., 2015; Wang M. J. et al., 2023).

Traditional Chinese Medicine (TCM) holds a distinctive position in the management of diseases that are challenging to treat. PSORI-CM01, a formulation comprising seven traditional Chinese herbs, demonstrates remarkable efficacy in the management of Psoriasis vulgaris (Wei et al., 2016). PSORI-CM01 is mainly composed of *Smilacis Glabrae Rhizoma* (*Smilax glabra Roxb.*, dried rhizome), *Sarcandrae Herba* (*Sarcandra glabra (Thunb.) Nakai*, dried whole plant), *Paeoniae Radix Rubra* (*Paeonia lactiflora Pall.* or *Paeonia veitchii Lynch*, dried root), *Mume Fructus* (*Prunus mume (Sieb.) Sieb. etZucc.*, dried nearly ripe fruit), *Arnebiae Radix* (*Arnebia euchroma (Royle) Johnst.* or *Arnebia guttata Bunge*, dried root), *Curcumae Rhizoma* (*Curcuma phaeocaulis Val., Curcuma kwangsiensis S.G.Lee et C.F.Liang* or *Curcuma wenyujin Y.H.ChenetC.Ling*, dried root), *Glycyrrhizae Radix Et Rhizoma* (*Glycyrrhiza uralensis Fisch., Glycyrrhiza inflata Bat.* or *Glycyrrhiza glabra L.,* dried root and rhizome), and the dosage of each herb is 55 g, 30 g, 12 g, 12 g, 9 g, 9 g, and 6 g. The chemical constituents of this formulation have been identified utilizing ultra-high liquid chromatography coupled with electrospray ionization hybrid linear trap quadrupole Orbitrap mass spectrometry (UHPLC-ESI-LTQ/Orbitrap-MS) (Chen et al., 2015). Nonetheless, the precise mechanisms underlying the therapeutic efficacy of PSRO-CM01 in the management of Psoriasis vulgaris remain to be comprehensively elucidated.

In the present study, the mechanisms of PSORI-CM01 in the treatment of psoriasis vulgaris were investigated. Psoriasis-like mice was induced by imiquimod (IMQ) inducing BALb/c mice, and psoriasis-like keratinocytes was constructed by IL-6 stimulation in HaCaT cells. These models were treated with different concentrations of PSORI-CM01, and then PASI scores, epidermal thickness, expression of miR-31 and Krt6 were determined. Moreover, psoriasis-like keratinocytes and psoriasis-like keratinocytes transfected with miR-31 mimics were treated with PSORI-CM01 respectively, and the expression of Krt6 was measured.

## 2 Materials and methods

### 2.1 Animals

Male BALB/c mice, aged between 6 and 8 weeks, were procured from Beijing Vitong Lihua Laboratory Animal Technology Co., Ltd. (Beijing, China). All experimental protocols were executed in strict compliance with the guidelines stipulated by the National Institutes of Health for the Care and Use of Laboratory Animals and received approval from the Animal Use Review Committee at Chongqing Hospital of Traditional Chinese Medicine. The psoriasis-like mice model was induced as previously described (Zhang et al., 2018). In brief, the dorsal hair of BALB/c mice was trimmed to an area measuring 1.5 × 1.0 cm, following which IMQ cream (Ming Xin Pharmaceutical Co. Ltd., Sichuan, China; dosage: 62.5 mg per mouse per day) was administered to the denuded skin for a duration of 7 consecutive days.

### 2.2 Cells

The HaCaT cell line, which is an immortalized line of human keratinocytes, was obtained from the American Type Culture Collection, situated in Manassas, Virginia, United States. The cells were cultured in Dulbecco’s Modified Eagle Medium (Gibco, California, United States), supplemented with 10% fetal bovine serum (Gibco, California, United States), devoid of any antibiotics, in accordance with previously established protocols (Zhang et al., 2018). The cell culture was maintained in a humidified atmosphere with 5% CO_2_ at a temperature of 37°C. Upon reaching 40%–60% confluence, the cells were exposed to IL-6 (CST, United States) at a concentration of 10 ng/mL, to establish psoriasis-like cell model.

### 2.3 Plant materials and preparation of PSORI-CM01

The PSORI-CM01 formula utilized in this study consisted of 55 g *Smilacis Glabrae Rhizoma* (Tu Fu Ling), 30 g *Sarcandrae Herba* (Zhong Jie Feng), 12 g *Paeoniae Radix Rubra* (Chi Shao), 12 g *Mume Fructus* (Wu Mei), 9 g *Arnebiae Radix* (Zi Cao), 9 g *Curcumae Rhizoma* (E Zhu), and 6 g *Glycyrrhizae Radix Et Rhizoma* (Gan Cao). All of the plant materials used in this study were of pharmacopoeia-grade and were obtained from Chongqing Qingxiang Pharmaceutical Co., Ltd. (Chongqing, China). The herbs were extracted via distilled water, and the resulting extracts were concentrated to volume of 29.91 mL.

For the mouse study, the high-dose PSORI-CM01 group received a daily oral gavage administration of 0.1 mL of the decoction. The medium-dose group was administered 0.05 mL of the decoction daily via oral gavage; this dose was diluted with physiological saline to achieve a final volume of 0.1 mL. Similarly, the low-dose PSORI-CM01 group received a daily oral gavage dose of 0.025 mL of the decoction, also diluted to a final volume of 0.1 mL using physiological saline. The positive reference drug group was administered methotrexate tablets (2.5 mg/tablet), prepared by dissolving one tablet in 12.92 mL of physiological saline. Mice in this group received 0.210 mL of this solution orally every 12 h, for a total of three consecutive administrations.

For preparation of serum with drugs, SD rats were given a daily oral gavage dose of 1.21 mL of the decoction. Following seven consecutive days of administration, blood was collected from the abdominal aorta and centrifuged to obtain drug-containing serum. This serum was then used to treat different intervention groups. The low-concentration PSORI-CM01 group received a final concentration of 5% drug-containing serum (combined with 10% blank serum). The medium-concentration group received a final concentration of 10% drug-containing serum (combined with 5% blank serum), while the high-concentration PSORI-CM01 group received a final concentration of 15% drug-containing serum.

### 2.4 Western blotting (WB) assay

The WB assay was conducted as previously described (Chen et al., 2019). In brief, the protein was extracted using RIPA Buffer (CST, Boston, United States), which comprises a cocktail of protease and phosphatase inhibitors (Beyotime, Shanghai, China). The quantification of protein concentration was executed utilizing the BCA Protein Assay kit (Beyotime, Shanghai, China). Equivalent amounts of proteins were subjected to electrophoretic fractionation utilizing 10% Sodium Dodecyl Sulfate Polyacrylamide Gel Electrophoresis (SDS-PAGE). Following this, the fractionated proteins were translocated onto Polyvinylidene Difluoride (PVDF) membranes (Millipore, Billerica, United States). The electrophoretic mobility and translocation of proteins were supervised utilizing the PageRuler Plus Prestained Protein Ladder (Thermo Scientific, Waltham, United States). The PVDF membranes were subjected to a blocking process with a 5% solution of non-fat dry milk for 1 h at room temperature. Following blocking, the membranes were subjected to wash for 5 min. Subsequently, the membranes were subjected to an overnight incubation at 4°C with primary antibodies, specifically mouse monoclonal anti-KRT 6 (1:500, Santa Cruz, Shanghai, China) and rabbit monoclonal anti-GAPD (1:2,000, CST, Boston, United States). The PVDF membranes were subjected to three washes, each lasting for 5 min. Post-washing, the membranes were subjected to an incubation period of 1 h at room temperature with secondary antibodies, specifically HRP-labeled goat anti-mouse IgG (H + L) (1:1,000, CST, Boston, United States) or HRP-labeled goat anti-rabbit IgG (H + L) (1:1,000, CST, Boston, United States). The detection of antibody binding was facilitated utilizing the Enhanced Chemiluminescence (ECL) Western blotting Detection System (Millipore, Billerica, United States).

### 2.5 Quantitative real-time reverse transcriptase–polymerase chain reaction (qRT-PCR) assay

Total RNA was extracted from both keratinocytes and skin biopsy specimens utilizing the Trizol reagent (Invitrogen, Shanghai, China). The quality of the isolated RNA was evaluated utilizing a NanoDrop spectrophotometer (ND-1000). The quantification of mature miR-31 was conducted following the guidelines provided in the manufacturer’s instruction manual. In summary, a quantity of 50 ng of the total RNA was utilized for the reverse transcription process. This was accomplished using the TaqMan microRNA Reverse Transcription Kit, along with miR-31 specific reverse transcription primers. Additionally, U6 snRNA (Applied Biosystems, Shanghai, China) was employed as an intrinsic control. The synthesized complementary DNA (cDNA) was subsequently subjected to analysis through qRT-PCR. This process utilized TaqMan probes that were specifically designed for miR-31 and U6 snRNA. Primers sequences for target genes as previous described (Yan et al., 2015). The relative abundance of miRNA was determined utilizing the comparative critical threshold (CT) methodology. The quantification of expression levels was standardized relative to the intrinsic U6 expression and computed utilizing the equation: expression = 2^−ΔΔCT^.

### 2.6 Transfection assay

To induce miR-31 overexpression in HaCaT cells, transfection with miR-31 mimics was performed utilizing the LipoRNAi™ reagent (Beyotime, Shanghai, China), and This procedure was conducted in strict adherence to the guidelines provided by the manufacturer. In summary, cells were distributed at a density of 2.5 × 10^5^ cells per well within a six-well plate. Upon reaching 75% confluence, transfection was performed. A total of 125 μL of DMEM without FBS, supplemented with miR-31 mimics and 4 μL of LipoRNAi reagent, was incubated for 20 min at room temperature. Subsequently, the mixture was added to the six-well plate with 1.75 mL of normal DMEM per well.

### 2.7 PASI scores of skin lesion

An objective evaluation system, grounded in the clinical Psoriasis Area and Severity Index (PASI), was established for the purpose of gauging the severity of psoriatic skin manifestations in murine models. The intensity of erythema, scales, and thickening was independently evaluated utilizing a scale that spanned from 0 to 4. Here, a score of 0 denoted the absence of symptoms, 1 represented mild symptoms, 2 corresponded to moderate symptoms, 3 signified pronounced symptoms, and 4 indicated extremely severe symptoms. The cumulative score, obtained by summing the individual scores for erythema, scales and thickening, was utilized to quantify the severity of the psoriatic lesions, with maximum possible score of 12.

### 2.8 Histological assay

Samples of murine dorsal skin were fixed in formalin for 48 h. Following this, the specimens were encased in paraffin, partitioned to a thickness of 6 μm, and subjected to staining with hematoxylin and eosin (HE) for the purpose of histological examination. The thickness of the epidermis was gauged from the basement membrane up to the stratum corneum. For the immunohistochemical (IHC) analysis, the expression of Krt6 in skin samples was evaluated using mouse anti-Krt6 monoclonal antibody (1:300, Santa Cruz, Shanghai, China), in accordance with the manufacturer’s instructions. The expression of Krt6 was quantified using histochemical scores (H scores). The H scores for Krt6 were assessed on a scale that spanned from 0 to 4. Here, a score of 0 denoted the absence of expression, 1 represented mild expression intensity, 2 corresponded to moderate expression intensity, 3 signified strong expression intensity, and 4 indicated extremely high expression intensity. All specimens or regions within each specimen were chosen employing a double-blind approach.

### 2.9 *In situ* hybridization assay

*In situ* hybridization assay was conducted on frozen tissue sections that had a thickness of 10 mm. In a concise manner, following a 10-min incubation period in an acetylation solution composed of 0.02 M Hydrochloric Acid (HCl), 1.3% Triethanolamine, and 0.25% Acetic Anhydride in Diethyl Pyrocarbonate-treated water at room temperature, the sections underwent treatment with Proteinase K (concentration of 5 mg/mL) in Phosphate-Buffered Saline (PBS) for a duration of 10 min. This was followed by a washing process and a prehybridization phase lasting 6 h. Hybridization with mmu-miR-31 5′-DIG and 3′-DIG-labelled miRCURY LNA detection probe (Exiqon, Beijing, China) was performed overnight at 50°C. After hybridization, the slides were washed with 5 × saline sodium citrate (SSC) buffer, followed by incubation in 0.2×SSC buffer for 1 h at temperature of 60°C. Probe binding was detected by incubating the sections with an anti-DIG-alkaline phosphatase antibody (1:2,000, Roche, Shanghai, China) overnight at 4°C. Visualization of the sections was achieved using NBT/BCIP ready-to-use tablets, in accordance with the manufacturer’s instructions.

### 2.10 Statistical analysis

All data pertinent to this study was subjected to analysis utilizing the GraphPad Prism software, version 9.5.1. The assessment of normality and homogeneity of variance was conducted utilizing the Shapiro-Wilk and Levene’s tests, respectively. A one-way analysis of variance (ANOVA), followed by Dunnett’s *post hoc* test, was employed for the comparison of multiple groups against control group. One-way ANOVA followed by Bonferroni *post hoc* tests was used for multiple comparisons between groups. Non-parametric data was analyzed utilizing the Mann-Whitney U test and Kruskal-Wallis test. Parametric data is presented as mean ± standard error of the mean (SEM), while non-parametric data is presented as median ± range. The determination of statistical significance was established at the following levels: *P < 0.05, **P < 0.01, ***P < 0.001 and ****P < 0.0001.

## 3 Results

### 3.1 PSORI-CM01 alleviates the clinical symptoms of psoriasis-like lesions in murine models

**FIGURE 1:**
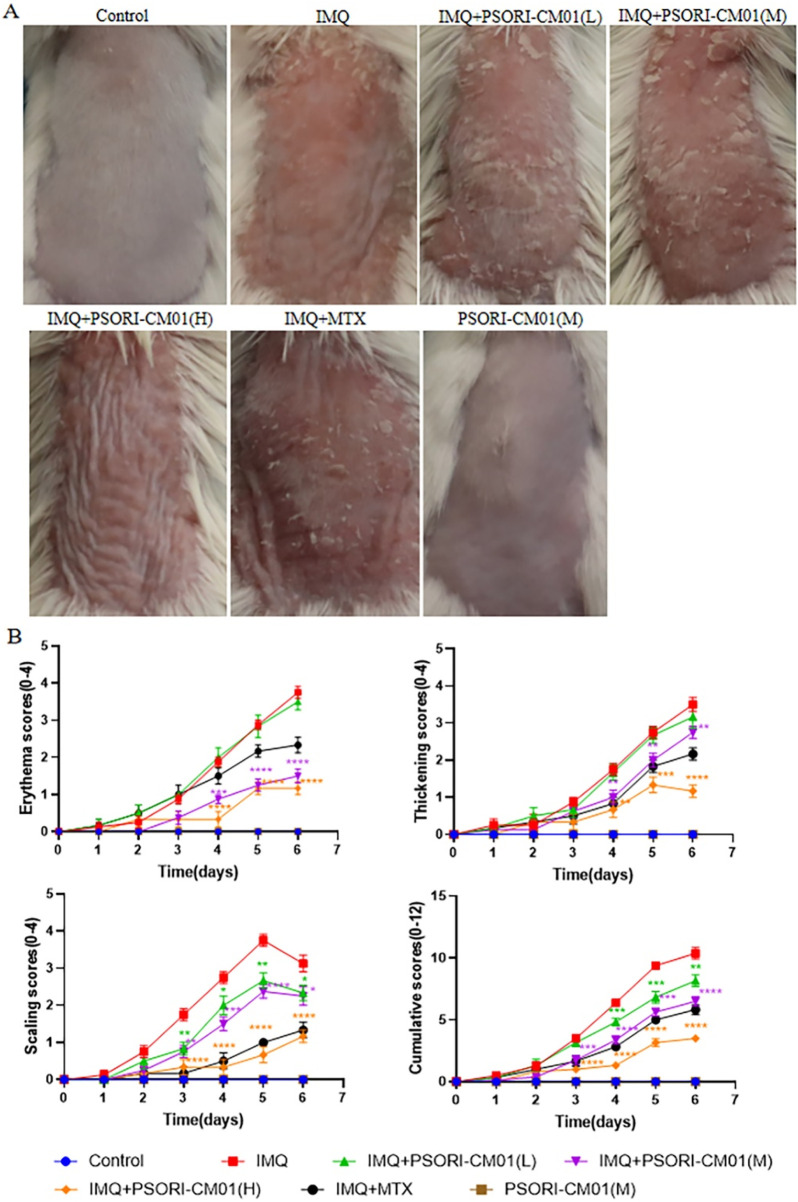
PSORI-CM01 alleviates the clinical symptoms of psoriasis-like lesions in murine models. Male BALB/c mice, aged between 6 to 8 weeks, were subjected to a daily topical regimen for a period of 7 days, involving the application of IMQ cream, either alone or in combination with PSORI-CM01, on their depilated dorsal skin. **(A)** Phenotypic presentation of the dorsal skin of the mice. **(B)** PASI scores of the skin lesions. *P < 0.05, **P < 0.01, ***P < 0.001, ****P < 0.0001, n = 3. MTX, Methotrexate (positive drug).

PSORI-CM01 is a formula composed of seven Chinese herbs. It is commonly utilized in the treatment of psoriasis vulgaris. To assess the efficacy of PSORI-CM01 in ameliorating psoriatic lesions, we administered it to psoriatic mice and monitored its clinical manifestations. The results demonstrated that PSORI-CM01 significantly improved the clinical manifestations of erythema, scales and thickening, and reduced the PASI scores in psoriatic mice (Figures 1A,B).

### 3.2 PSORI-CM01 reduces epidermal thickness in psoriasis-like lesions in murine models

**FIGURE 2:**
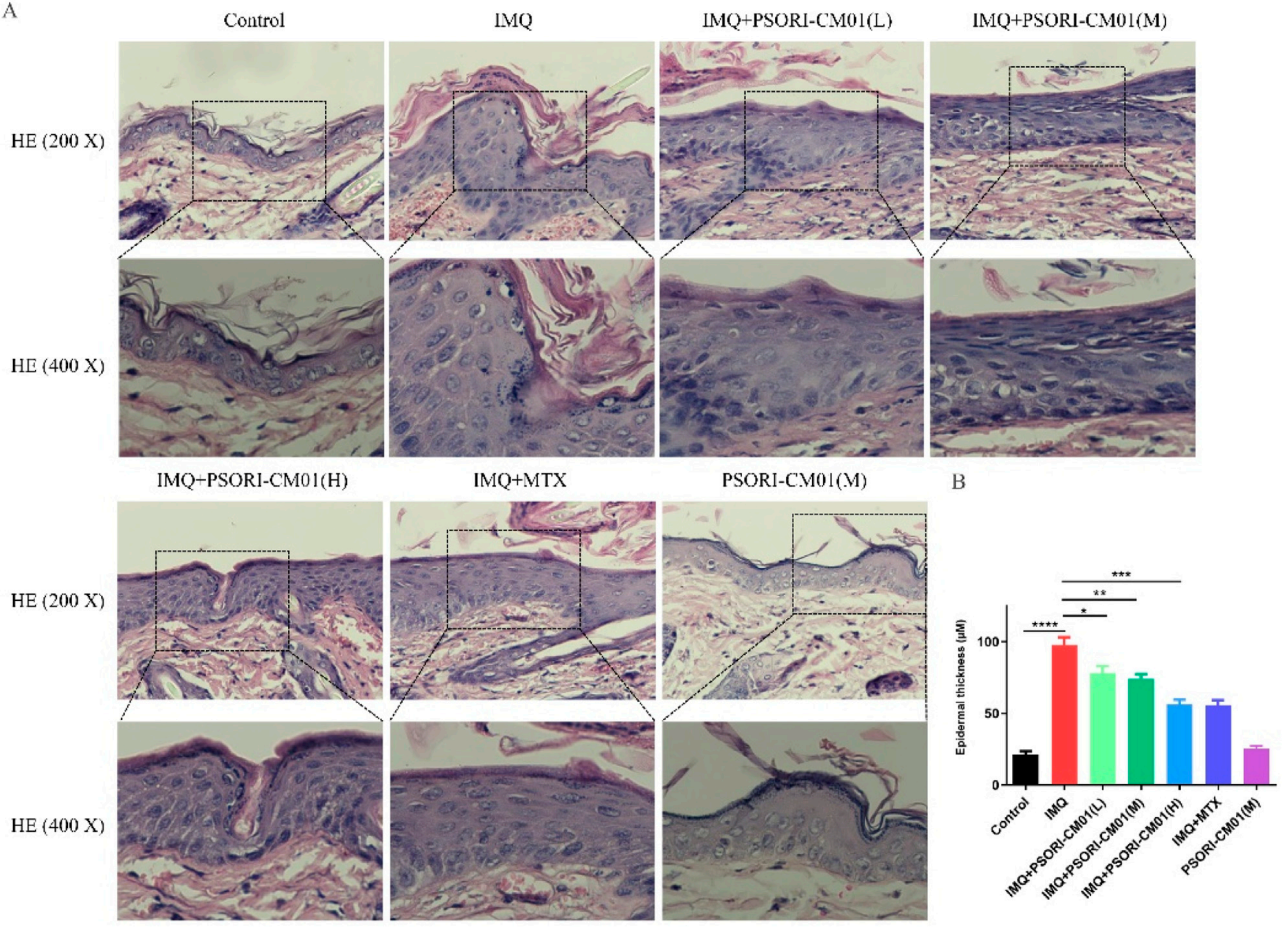
PSORI-CM01 reduces epidermal thickness in psoriasis-like lesions in murine models. Male BALB/c mice, aged between 6 to 8 weeks, were subjected to a daily topical regimen for a period of 7 days, involving the application of IMQ cream, either alone or in combination with PSORI-CM01, on their depilated dorsal skin. **(A)** Epidermal thickness was assessed using HE. **(B)** Statistical analysis was performed on the epidermal thickness data obtained from the results in **(A)**. *P < 0.05, **P < 0.01, ***P < 0.001, ****P < 0.0001, n = 3.

Hyperplasia of the epidermis is a principal pathological alteration observed in the cutaneous lesions of psoriasis vulgaris (Chen et al., 2021; Hao et al., 2021). Therefore, we utilized HE staining to assess the impact of PSORI-CM01 on epidermal thickness. Compared to control group, the epidermis of IMQ-induced psoriasis-like mice exhibited significant thickening. PSORI-CM01 significantly reduced the thickness of the epidermis in psoriasis-like mice in dose-dependent manner (Figures 2A,B). The efficacy of high-dose PSORI-CM01 in reducing epidermal thickness was comparable to methotrexate (MTX), a commonly used medication for psoriasis vulgaris. Medium-dose PSORI-CM01 had no effect on the epidermal thickness of normal mice.

### 3.3 PSORI-CM01 inhibits expression of miR-31 in psoriasis-like lesions in murine models

**FIGURE 3:**
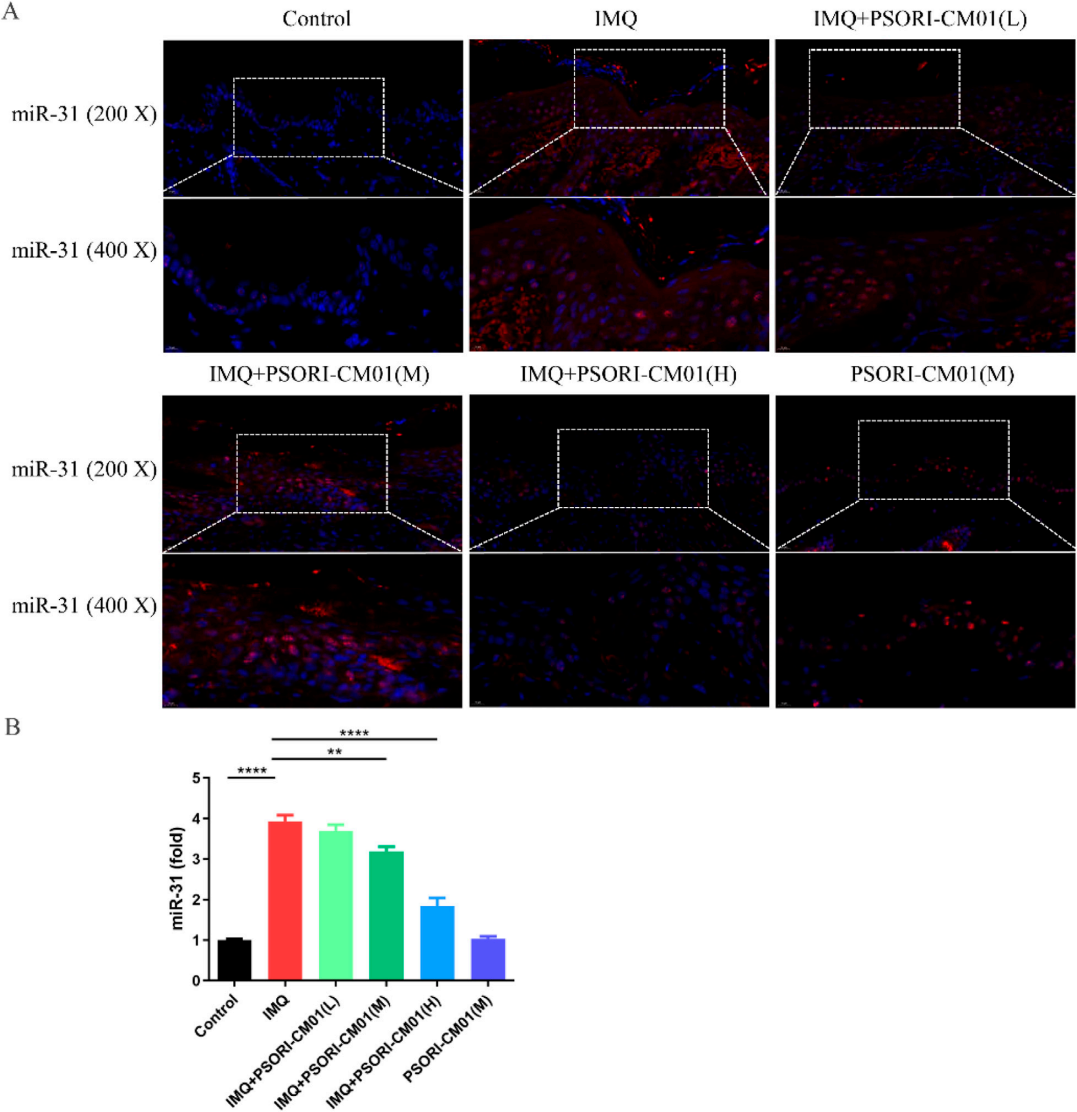
PSORI-CM01 inhibits expression of miR-31 in psoriasis-like lesions in murine models. Male BALB/c mice, aged between 6 to 8 weeks, were subjected to a daily topical regimen for a period of 7 days, involving the application of IMQ cream, either alone or in combination with PSORI-CM01, on their depilated dorsal skin. **(A)** Expression of miR-31 was quantified using *in situ* hybridization. **(B)** Expression of miR-31 was quantified using qRT-PCR. **P < 0.01, ****P < 0.0001, n = 3.

MiRNAs are diminutive, non-coding RNA molecules that exhibit a high degree of conservation across various species. They play a pivotal role in the regulation of gene expression at the post-transcriptional stage. MiR-31 exhibits overexpression in the cutaneous lesions of psoriasis vulgaris, whereas it is expressed at lower levels in the skin of healthy individuals (Yan et al., 2015; Zhang et al., 2015; Xu et al., 2013). To investigate the effect of PSORI-CM01 on miR-31 expression, we employed *in situ* hybridization. Compared to the control group, the results showed the expression of miR-31 was significantly upregulated in the skin lesions of psoriasis-like mice. However, treatment with different concentrations of PSORI-CM01 resulted in significant downregulation of miR-31 expression in psoriasis-like mice (Figure 3A). Furthermore, we used qRT-PCR to quantify miR-31 expression and found that the results were consistent with those obtained through *in situ* hybridization (Figure 3B).

### 3.4 PSORI-CM01 inhibits Krt6 expression in psoriasis-like lesions in murine models

**FIGURE 4:**
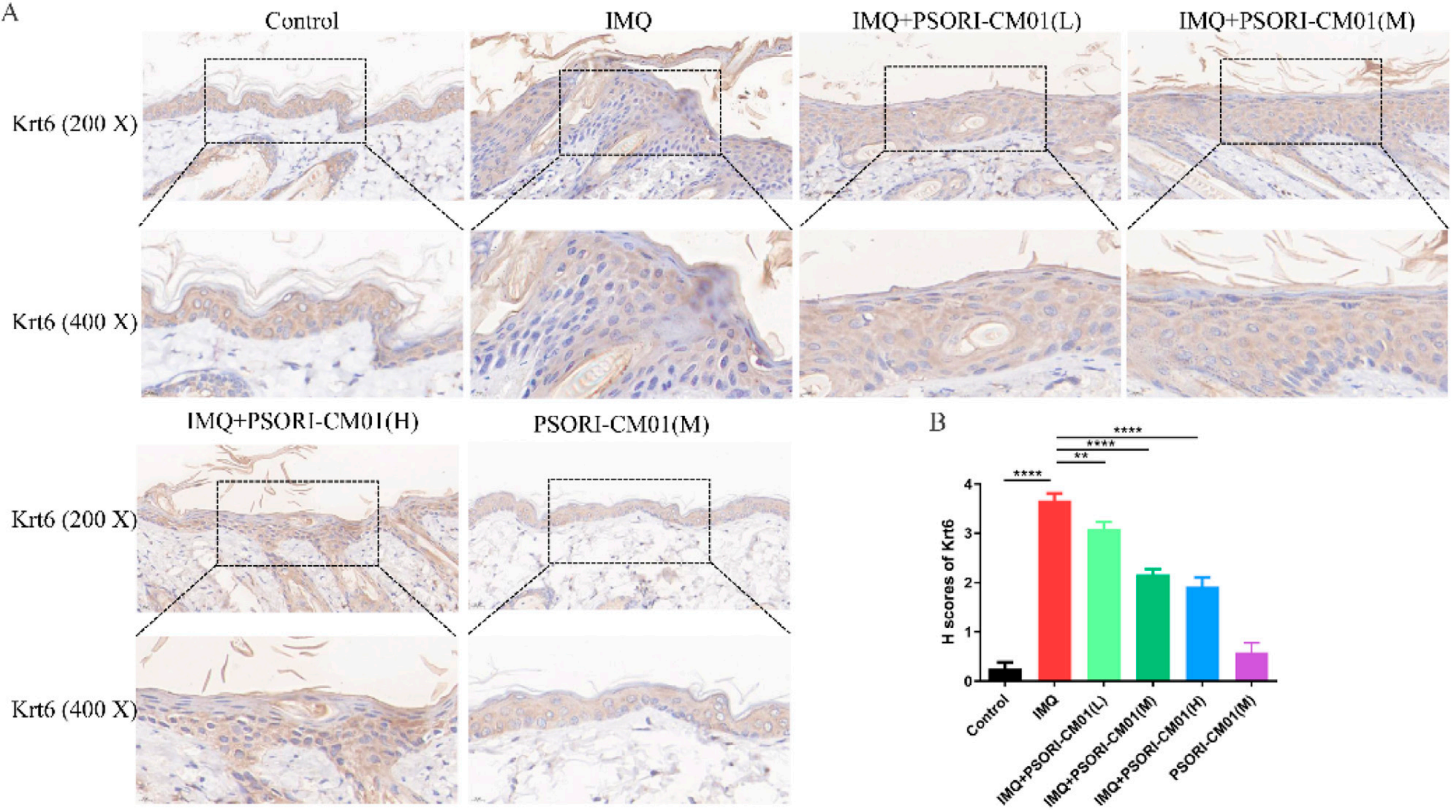
PSORI-CM01 inhibited Krt6 expression in psoriasis-like lesions in murine models. Male BALB/c mice, aged between 6 to 8 weeks, were subjected to a daily topical regimen for a period of 7 days, involving the application of IMQ cream, either alone or in combination with PSORI-CM01, on their depilated dorsal skin. **(A)** Expression of Krt6 was assessed using IHC. **(B)** Statistical analysis was performed on the Krt6 expression data obtained from the results in **(A)**. **P < 0.01, ****P < 0.0001, n = 3.

Keratins are major component of keratinocytes and play an important role in both adaptive and innate immunity. Krt6 regulates cell proliferation, keratinization and inflammatory responses. Hyperproliferation of Krt6 is a hallmark pathological feature in patients with psoriasis vulgaris. In murine models of psoriasis, Krt6 is overexpressed in psoriatic lesions, and suppressing Krt6 expression ameliorates the severity of psoriatic-like lesions (Qiao et al., 2022; Yang et al., 2017; Zhang et al., 2019). Krt6 is considered as a key diagnostic marker for psoriasis. To evaluate the anti-psoriatic efficacy of PSORI-CM01, we utilized IHC assay to assess its impact on Krt6 expression. In our study, we observed significant upregulation of Krt6 expression in psoriasis-like mice. However, treatment with PSORI-CM01 notably suppressed Krt6 expression in the mice (Figures 4A,B).

### 3.5 PSORI-CM01 inhibits expression of Krt6 through miR-31

**FIGURE 5:**
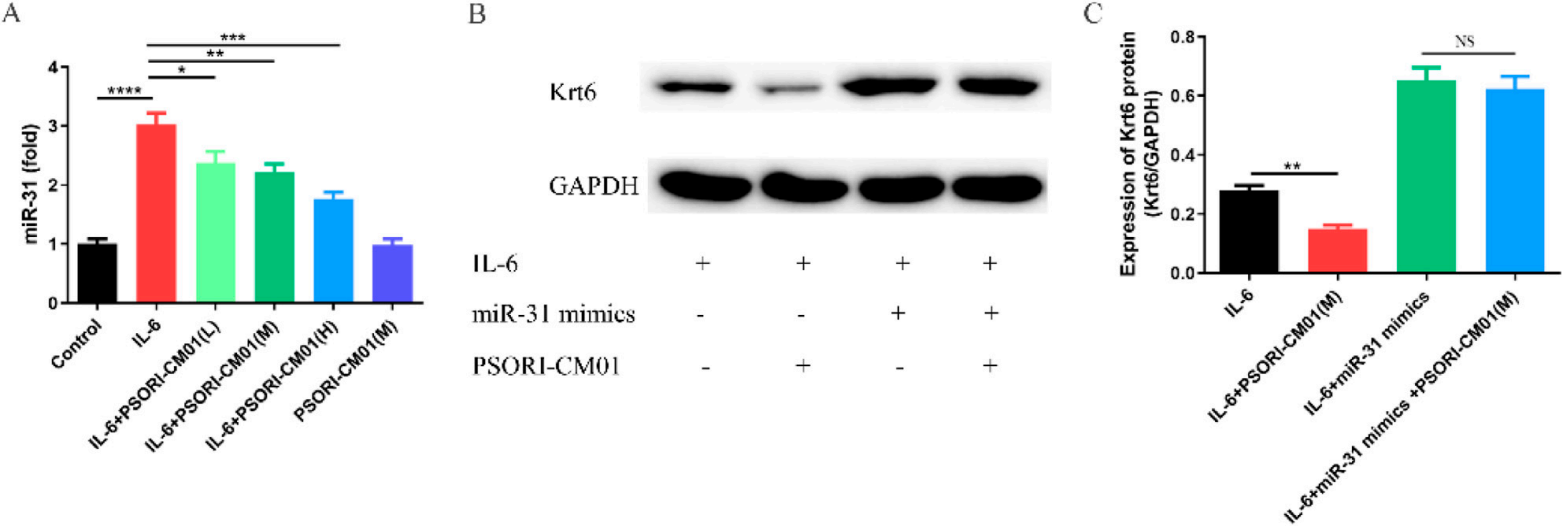
PSORI-CM01 inhibits expression of Krt6 through miR-31. **(A)** HaCaT cells were treated with IL-6 in the presence or absence of PSORI-CM01 for 24 h. The expression of miR-31 was quantified using qRT-PCR. **(B)** HaCaT cells induced with IL-6 were transfected with or without miR-31 mimics and subsequently treated with PSORI-CM01. **(C)** The expression of Krt6 was assessed using WB. *P < 0.05, **P < 0.01, ***P < 0.001, ****P < 0.0001, n = 3.

*In vivo*, we discovered that PSORI-CM01 inhibited the expression of miR-31 and Krt6, and it improved psoriasis-like lesions. Subsequently, we evaluated the effect of PSORI-CM01 on miR-31 and Krt6 *in vitro*. We induced HaCaT cells with IL-6 (10 ng/mL) for 24 h to build psoriasis-like cellular model. PSORI-CM01 was applied at various concentrations to intervene in these psoriasis-like keratinocytes. The expression of miR-31 and Krt6 proteins was quantified using qRT-PCR and WB assays. In this study, we found that the expression of miR-31 and Krt6 was significantly upregulated in IL-6-induced psoriasis-like keratinocytes; however, treatment with PSORI-CM01 significantly decreased the expression of both miR-31 and Krt6 in this psoriasis-like keratinocytes (Figures 5A–C).

This study has discovered that PSORI-CM01 inhibited the expression of miR-31 and Krt6 proteins *in vivo* and *in vitro*. Therefore, we hypothesized that PSORI-CM01 might inhibit Krt6 protein expression through miR-31. To investigate this hypothesis, we treated psoriasis-like keratinocytes and these cells transfected with miR-31 mimics with PSORI-CM01 respectively, and we measured the expression of Krt6 protein. We found that PSORI-CM01 significantly decreased the expression of Krt6 protein in psoriasis-like keratinocytes; however, transfection with miR-31 mimics reversed the inhibitory effect of PSORI-CM01 on Krt6 protein (Figures 5B,C). These results suggested that PSORI-CM01 might inhibit Krt6 expression through miR-31.

## 4 Discussion

PSORI-CM01, a compound formula in Chinese medicine, has been shown to be effective in treating psoriasis vulgaris. However, the mechanisms of action of PSORI-CM01 have not been fully understood. Our research on the mechanism of PSORI-CM01 in treating psoriasis vulgaris *in vivo* and *in vitro* suggests that PSORI-CM01 may improve psoriatic lesions by inhibiting Krt6 dependent of miR-31.

Psoriasis vulgaris is a common but serious disease that can occur at any age and can significantly impair the patient’s quality of life. In 2014, the WHO recognized psoriasis as a serious disease and called on governments worldwide to pay attention to this condition. Psoriasis vulgaris is a complex disease that affects the skin and nails and is accompanied by numerous comorbidities. Cutaneous manifestations present as localized or generalized, symmetrically distributed, well-demarcated erythematous papules and plaques and typically covered with scales. These lesions can cause pruritus, burning and pain. Between 1.3% and 34.7% of patients with psoriasis vulgaris develop psoriatic arthritis (Sobec et al., 2023). Psoriatic arthritis is a chronic inflammatory arthritis that can lead to joint deformation and even disability. Moreover, nail changes occur in 4.2%–69% of psoriasis vulgaris cases. Patients with psoriasis vulgaris are at increased risk for cardiovascular disease, metabolic disease, diabetes and other conditions. Altogether, psoriasis vulgaris patients impose severe physical, emotional and social burden. The etiology of psoriasis vulgaris remains elusive, although genetic predisposition and immune dysregulation are implicated. Current treatment strategies aim to control symptoms, but no cure for psoriasis vulgaris exists.

Chinese herbal, which has been used for at least 2,000 years, is one of the important methods of treating diseases in Chinese medicine. Today, Chinese herbal still plays an important role in controlling and treating diabetes, coronary heart disease, hypertension and other conditions (Cao et al., 2021; Oduro et al., 2020; Wang et al., 2022). PSORI-CM01 is an optimized version of the Chinese medicine formula Yinxieling, which is originally developed by Guowei Xuan, a national Chinese medicine expert specializing in dermatology. PSORI-CM01 consists of seven medicinal herbs, including Smilacis Glabrae Rhizoma (Tu Fu Ling), Sarcandrae Herba (Zhong Jie Feng), Paeoniae Radix Rubra (Chi Shao), Mume Fructus (Wu Mei), Arnebiae Radix (Zi Cao), Curcumae Rhizoma (E Zhu) and Glycyrrhizae Radix Et Rhizoma (Gan Cao). Using UHPLC-ESI-LTQ/Orbitrap-MS, it was found that PSORI-CM01 contains 4 classes (organic acids, phenolic acids, flavonoids and terpenoids) and 108 compounds. Of these 108 compounds, 14 have been reported to exhibit multiple biological activities, including anti-inflammatory, immunoregulatory and anti-tumor properties, which are attributed to the treatment of psoriasis vulgaris (Chen et al., 2015). Among the fourteen compounds investigated, several have demonstrated relevance to psoriasis research: paeoniflorin modulates psoriasis-associated pathways, including the MAPKs/NF-κB pathway, PI3K/Akt/mTOR pathway, and JAK2/STAT3 pathway; liquiritin alleviates psoriasis through the modulation of the NF-κB pathway, AP-1 transcription factor, and the YY1/RBP3 axis; neoisoastilbin has been reported to inhibit the NF-κB/NLRP3 inflammasome pathway, which is also overexpressed in psoriasis; Glycyrrhizic acid significantly reduces the secretion of TNF-α, IL-12, IL-17, and IL-23 in psoriatic-like skin lesions; furthermore, neoastilbin, engeletin, and isoengeletin, components of the herbal formula PSORI-CM01, have been identified as active constituents in various herbal preparations used for psoriasis treatment (Chen et al., 2020; Deng et al., 2024; Wang Y. et al., 2023; Zhang and Wei, 2020; Zhang et al., 2023). Three dosage forms of PSORI-CM01 (tablet, granules and decoction) were manufactured and utilized in clinical setting at the Guangdong Provincial Hospital of Chinese Medicine. PSORI-CM01 has shown significant clinical efficacy in the treatment of psoriasis vulgaris. In this study, PSORI-CM01 significantly ameliorated the symptoms of psoriasis-like mice, including erythema, scales and thickening, as well as reducing PASI scores and epidermal thickness (Figure 1). Here, the anti-psoriatic effects of PSORI-CM01 were consistent with its clinical efficacy.

MiRNA is a category of non-coding, single-stranded RNA molecules, typically comprising approximately 22 nucleotides in length, which are encoded by intrinsic genes. They participate in post-transcriptional gene regulation in animals and plants. To date, over 250 miRNAs have been identified as being aberrantly expressed in psoriasis vulgaris tissue. Furthermore, 49 of these dysregulated miRNAs in psoriasis vulgaris have confirmed mRNA targets with established biological functions in cutaneous tissue (Jiang et al., 2023). Hyper-proliferation of keratinocytes is one of the most significant pathological features of psoriasis vulgaris. MiR-31 has been found to be overexpressed in psoriasis vulgaris skin and plays a role in regulating keratinocyte proliferation (Hawkes et al., 2016). The research by Xu et al. demonstrated that NF-κB signaling upregulates miR-31 expression, contributing to keratinocyte hyper-proliferation in psoriasis vulgaris (Xu et al., 2013). Yan et al. demonstrated that through the suppression of protein phosphatase 6, miR-31 facilitates the progression of the cell cycle, thereby promoting the proliferation of epidermal cells (Yan et al., 2015). Additionally, Shi et al. confirmed that miR-31 regulates Rasa1, Spred1, Spred2 and Spry4 to enhance keratinocyte proliferation (Shi et al., 2018). In our study, we discovered that PSORI-CM01 significantly inhibited the expression of miR-31, reduced the proliferation of epidermal keratinocytes and improved the lesions associated with psoriasis vulgaris.

Keratin is a fibrous structural protein that is present in hair, nails and epidermis. To date, over 54 mammalian keratins have been discovered, accounting for 30%–80% of total protein. In the epidermis, keratins are the primary constituents of the epithelial cytoskeleton. Keratins, while traditionally acknowledged for their crucial role in preserving skin integrity, have recently been identified as potent modulators of a broad spectrum of cellular processes. These include, but are not limited to, cell proliferation, migration, differentiation, and responses to inflammatory and immune stimuli. Mutations or abnormal expression of keratin proteins have been linked to various dermatological conditions, including psoriasis, bullous disorders and neoplasms (Elango et al., 2018). In normal interfollicular epidermis, the expression of Krt5 and Krt14 is recognized as characteristic markers of basal keratinocytes, while the expression of Krt1 and Krt10 in suprabasal keratinocytes reflects an early stage of differentiation. In contrast, the upregulation of Krt6 in keratinocytes is indicative of a state of heightened activation and proliferation, typically observed under pathological conditions (Coulombe, 2017; Ho et al., 2022). Recent research has indicated that the expression of Krt6 is significantly upregulated in the skin lesions of patients with psoriasis vulgaris. This elevation of Krt6 plays a pivotal role in the regulation of keratinocyte proliferation, thereby contributing to the pathophysiology of the disease. As such, Krt6 is generally considered as a biomarker and potential therapeutic target for psoriasis vulgaris (Gao et al., 2020; Ren et al., 2022). In our study, we observed marked upregulation of Krt6 expression in psoriasis-like mice and psoriasis-like keratinocytes. However, treatment with PSORI-CM01 at various doses significantly decreased Krt6 expression in the psoriasis-like model. These findings suggested that the anti-psoriatic effect of PSORI-CM01 might be mediated, at least in part, by its ability to modulate Krt6 expression.

We found that PSORI-CM01 alleviated the clinical characteristics of erythema, scales and thickening in psoriasis-like mice, reduced PASI scores and decreased epidermal thickness. Additionally, PSORI-CM01 inhibited the expression of miR-31 and Krt6. Therefore, we hypothesized that the anti-psoriatic effect of PSORI-CM01 may be mediated by the regulation of Krt6 through miR-31. To explore this hypothesis, psoriasis-like keratinocytes and those cells transfected with miR-31 mimics were treated with PSOR-CM01. Subsequently, the expression of Krt6 was assessed. We found that PSORI-CM01 significantly inhibited Krt6 expression in psoriasis-like keratinocytes; However, this suppressive effect of PSORI-CM01 on Krt6 was counteracted by miR-31 mimics. These results suggested that the amelioration of psoriasis-like lesions by PSRO-CM01 might be mediated by the regulation of Krt6 through miR-31.

## 5 Conclusion

**FIGURE 6:**
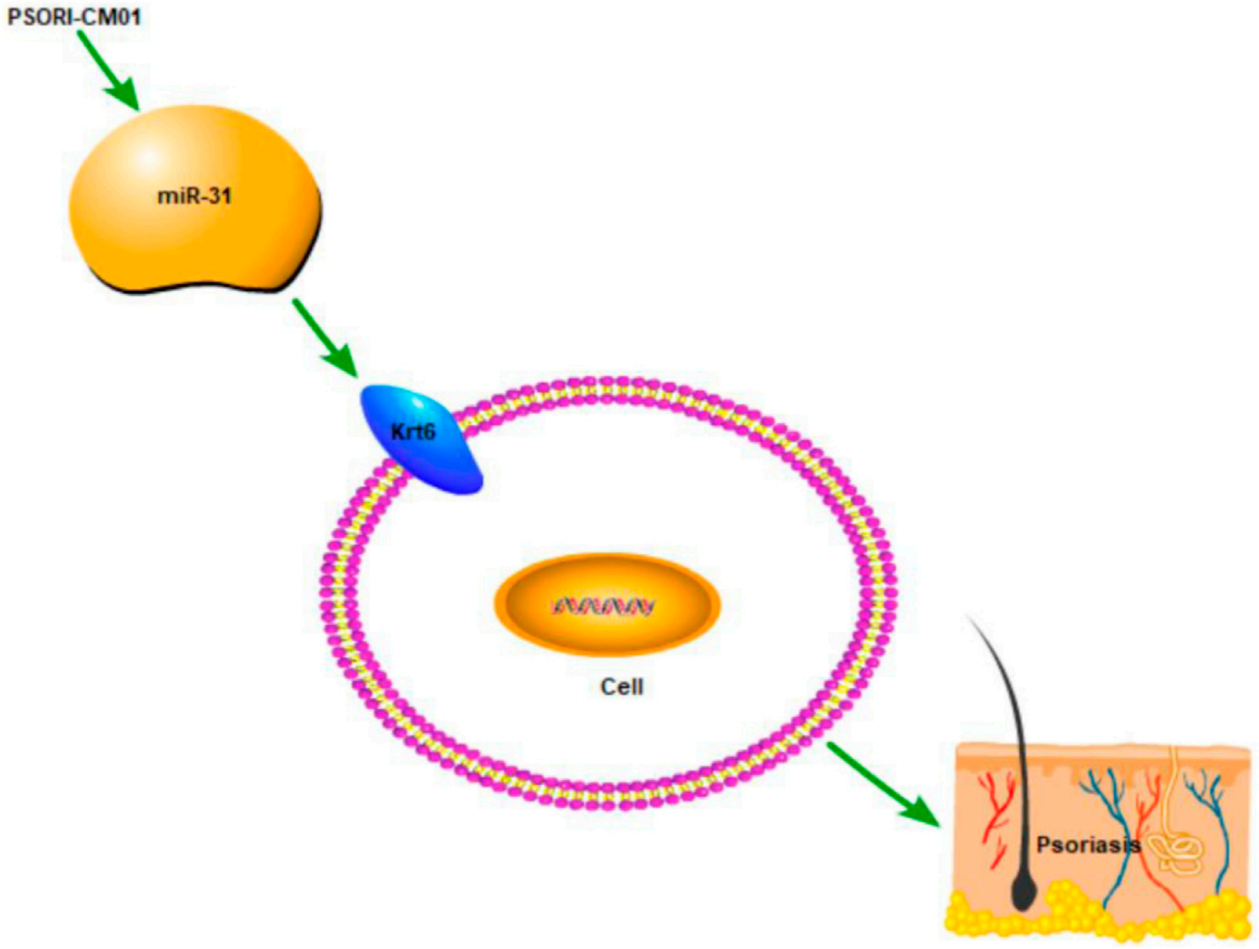
Schematic diagram of the anti-psoriasis mechanism of PSORI-CM01.

Psoriasis vulgaris is a common but severe disease. PSRO-CM01 is an effective prescription for the treatment of psoriasis vulgaris in clinical practice. In this study, we found that PSORI-CM01 might improve psoriasis-like lesions by Krt6 dependent of miR-31 (Figure 6). This research will help to understand the anti-psoriatic effect of PSORI-CM01 and promote its clinical application.

## Data Availability

The original contributions presented in the study are included in the article/Supplementary Material, further inquiries can be directed to the corresponding author.

## Abbreviations

IMQ, Imiquimod; IL-6, Interleukin 6; MiR-31, MicroRNA 31; Krt6, Keratinocyte 6; PASI, Psoriasis Area and Severity Index; WB, Western blotting; IHC, immunohistochemical; HE, Hematoxylin and Eosin.
