# Supplementary material for: MicroRNA-31 mediated inhibition of keratin 6 by PSORI-CM01: a novel approach to psoriasis amelioration
Source: Front Chem. 2025 Jul 18;13:1636529. doi: 10.3389/fchem.2025.1636529 (PMC12313620; doi:10.3389/fchem.2025.1636529)
Supplement: Supplementary file 1 [file Table1.pdf]

MicroRNA-31 Mediated Inhibition of Keratin 6 by PSORI-CM01: A Novel Approach to Psoriasis Amelioration

Table S1

Table S1 Primer sequence of mir-31 and U6 in qRT-PCR

qRT-PCR primer	Primer sequence
miR-31 Forward Sequence	GGCAAGATGCTGGCATAG
miR-31 Reverse Sequence	GAACATGTCTGCGTATCTC
U6 snRNA Forward primer	GCTTCGGCAGCACATATACTAAAAT
U6 snRNA Reverse primer	CGCTTCACGAATTTGCGTGTCAT

Table S2

Table S2 Primer sequence of mir-31, in situ hybridization

Probe name	Primer sequence
mir-31-5p	CAGCTATGCCAGCATCTTGCCT
