# Supplementary material for: MicroRNA-31 mediated inhibition of keratin 6 by PSORI-CM01: a novel approach to psoriasis amelioration
Source: Front Chem. 2025 Jul 18;13:1636529. doi: 10.3389/fchem.2025.1636529 (PMC12313620; doi:10.3389/fchem.2025.1636529)
Supplement: Supplementary file 2 [file DataSheet1.pdf]

MicroRNA-31 Mediated Inhibition of Keratin 6 by PSORI-CM01: A Novel Approach to Psoriasis Amelioration

Figure S1

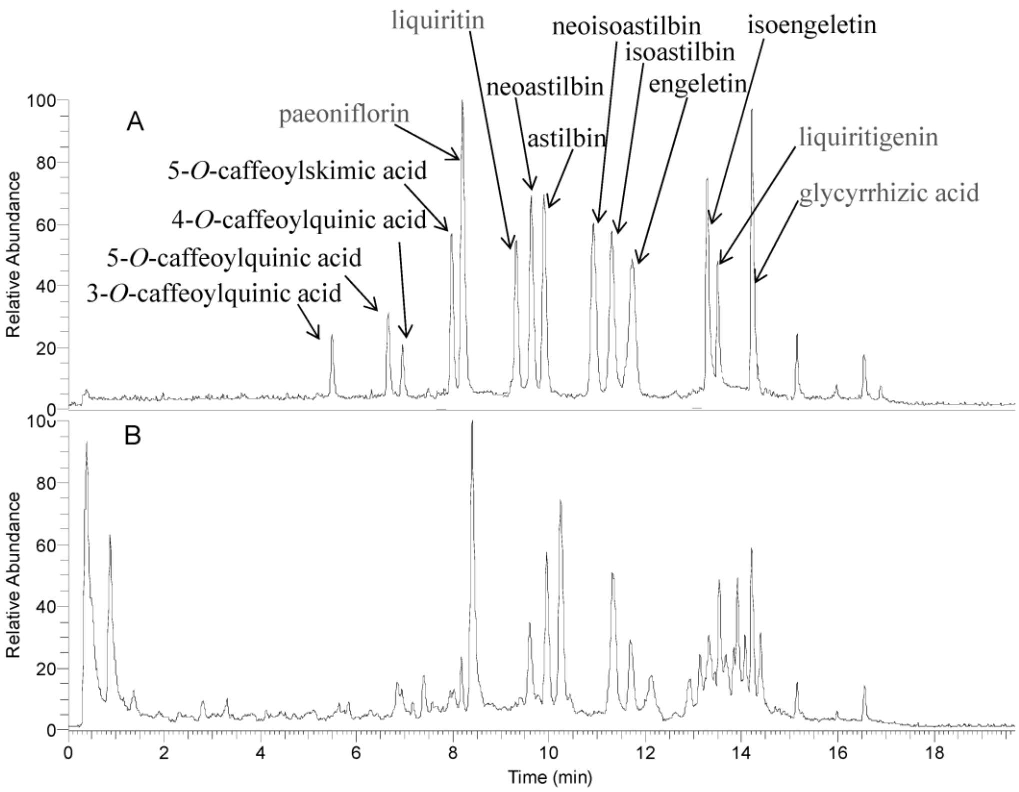

Figure S1. UHPLC-(-) ESI-MS total ion chromatograms of a mixture of PSORI-CM01 (B).

*Figure S1 is from "*Identification and quantitative characterization of PSORI-CM01, a Chinese medicine formula for psoriasis therapy, by liquid chromatography coupled with an LTQ Orbitrap mass spectrometer*"(DOI: 10.3390/molecules20011594)

Table S1

Table S1 Primer sequence of mir-31 and U6 in qRT-PCR

qRT-PCR primer	Primer sequence
miR-31 Forward Sequence	GGCAAGATGCTGGCATAG
miR-31 Reverse Sequence	GAACATGTCTGCGTATCTC
U6 snRNA Forward primer	GCTTCGGCAGCACATATACTAAAAT
U6 snRNA Reverse primer	CGCTTCACGAATTTGCGTGTCAT

Table S2

Table S2 Primer sequence of mir-31, in situ hybridization

Probe name	Primer sequence
mir-31-5p	CAGCTATGCCAGCATCTTGCCT
